# Empowering Women’s PrEP Choices: Qualitative Insights into Long-Acting PrEP Preferences and Decision-Making during Pregnancy and Breastfeeding in South Africa and Botswana

**DOI:** 10.1007/s10461-025-04856-y

**Published:** 2025-08-29

**Authors:** Jenny Chen-Charles, Lindsey De Vos, Prisca Vundhla, Avuyonke Gebengu, Elzette Rousseau, Linda-Gail Bekker, Remco Peters, Aamirah Mussa, Chelsea Morroni, Elona Toska, Chibuzor M. Babalola, Jeffrey D. Klausner, Dvora Joseph Davey

**Affiliations:** 1 Desmond Tutu HIV Centre, University of Cape Town, Cape Town, South Africa; 2 Department of Social Policy and Intervention, University of Oxford, Oxford, UK; 3 Desmond Tutu Health Foundation, Cape Town, South Africa; 4 Foundation for Professional Development, East London, South Africa; 5 Department of Medical Microbiology, University of Pretoria, Pretoria, South Africa; 6 Botswana Harvard Health Partnership, Gaborone, Botswana; 7 Usher Institute, The University of Edinburgh, Edinburgh, UK; 8 Centre for Social Science Research, University of Cape Town, Cape Town, South Africa; 9 Department of Population and Public Health Sciences, Keck School of Medicine, University of Southern California, Los Angeles, USA; 10 Department of Epidemiology and Biostatistics, School of Public Health, University of Cape Town, Cape Town, South Africa; 11 Division of Infectious Diseases, Geffen School of Medicine, University of California, Los Angeles, USA

**Keywords:** Pre-exposure prophylaxis, PrEP, Pregnant and breastfeeding women, Adolescent girls and young women, Long-acting PrEP, HIV prevention, Sub-Saharan africa

## Abstract

Pregnant and breastfeeding women (PBW) are at heightened risk of HIV acquisition. We aimed to explore PBW’s preferences of various long-acting PrEP modalities. In-depth interviews were conducted with PBW at three sites: Cape Town, East London (South Africa), and Gaborone (Botswana). We conducted thematic analysis, guided by the Health Belief Model, to examine participants’ HIV risk perception during pregnancy and breastfeeding, their perceived individual-level facilitators and barriers of each PrEP modality, and the support needed by participants for PrEP uptake and continuation. 40 participants were interviewed, aged 18–39 years (mean 27 years), including 13 adolescent girls and young women (AGYW; 18–24 years). Participants were either pregnant (*n* = 20) or breastfeeding (*n* = 20). Perceived HIV risk during pregnancy motivated PrEP use. Long-acting methods were preferred over daily pills for convenience and perceived reliability, with injectables most favoured due to familiarity with contraceptive injections. However, concerns about pain and side effects persisted. Monthly oral PrEP was seen as easier than daily pills but raised similar concerns about adherence. Implants were met with fear and mistrust, often rooted in negative contraceptive experiences, though some valued their long-term protection. The vaginal ring was least acceptable due to unfamiliarity and discomfort with insertion. Participants emphasised the importance of external support (e.g. reminders), privacy and discretion, and community transparency to support PrEP adherence and reduce stigma. Addressing barriers – especially concerns about the safety and effectiveness of new modalities and challenges around adherence – could help improve strategies to better assist PBW in utilising long-acting PrEP modalities.

**Supplementary Information:**

The online version contains supplementary material available at 10.1007/s10461-025-04856-y.

## Introduction

In sub-Saharan Africa (SSA), women, particularly adolescent girls and young women (AGYW), account for 62% of new HIV infections [1]. Pregnant and breastfeeding women (PBW) are at heightened risk of HIV acquisition, due to biological factors and behavioural changes during this period, such as hormonal shifts and immune system changes, which, alongside other behavioural factors, may increase susceptibility. Incident HIV infection during pregnancy and postpartum, in turn increases the risk of vertical transmission [2, 3] Pre-exposure prophylaxis (PrEP) is a highly effective HIV prevention product, and its introduction has been crucial in response to the urgent need to reduce HIV acquisition. However, adherence to daily oral PrEP is particularly challenging for women, especially during the transitional phases of pregnancy and postpartum, when factors such as side effects, the demands of infant care, and fluctuating sexual activity can disrupt consistent use [4–6]. Structural barriers such as limited healthcare access, sociocultural beliefs, and weak service integration within health systems, often make it difficult for PBW to access PrEP [7–12]. In addition, a broader set of psychosocial and relational factors may shape adherence behaviours and product preferences in this group, including depression and anxiety, intimate partner violence (IPV) or other forms of gender-based violence (GBV), stigma, and a lack of support from male partners, family, peers, or the broader community [6, 13, 14]. These factors may interact with structural inequalities and normative expectations of motherhood to compound risk or reduce agency over HIV prevention choices.

**Fig. 1:**
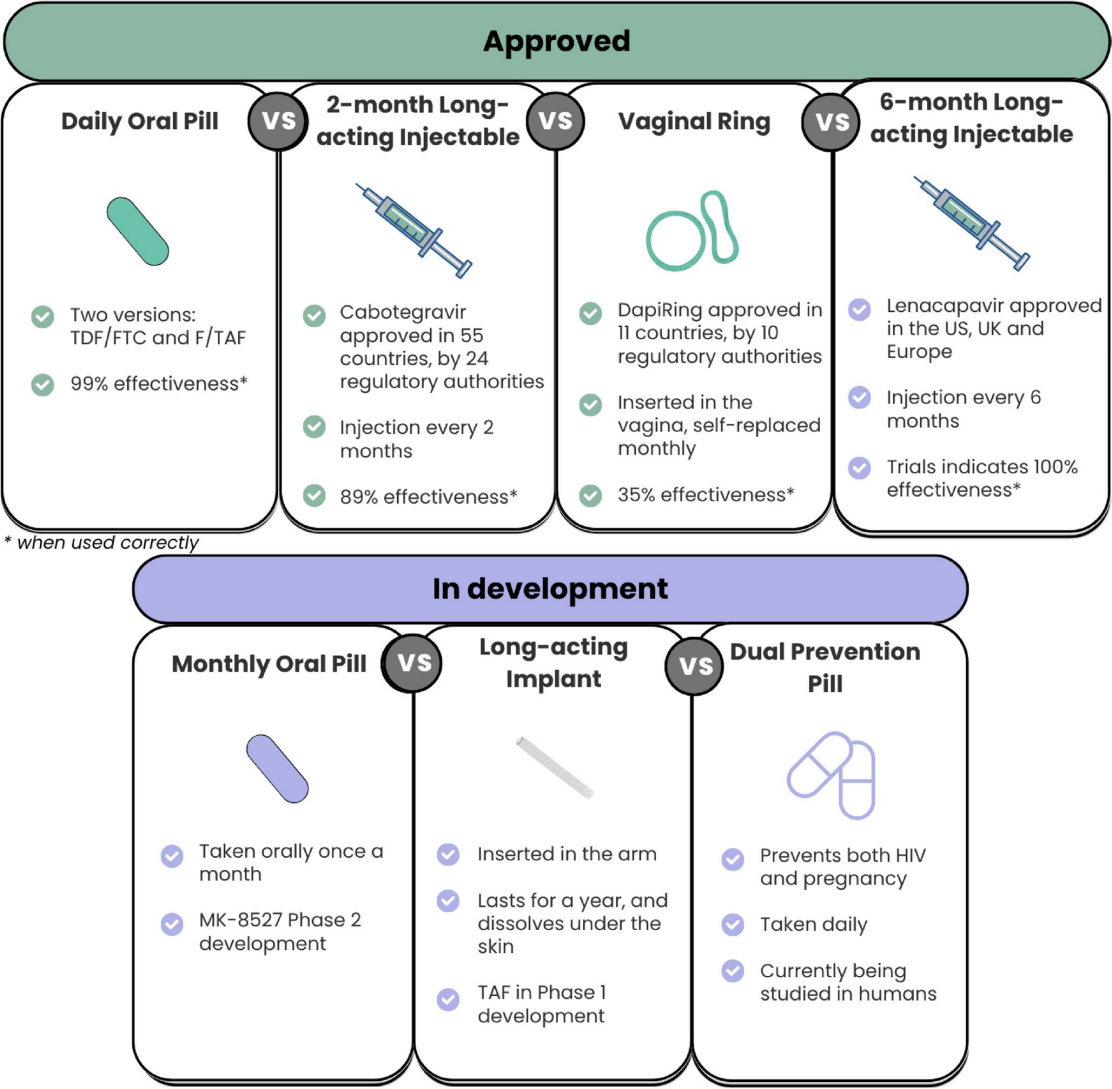
PrEP products approved and in development, information from:[59–61]

Several new long-acting PrEP modalities have been developed, are in development, or under consideration. These include monthly oral pills, long-acting injectables PrEP (such as Cabotegravir and Lenacapavir), the Dapivirine vaginal ring, monthly/infrequent oral pills, and the PrEP implants (see Fig. 1 for details) [15–19]. Alternative options offer different dosing schedules and delivery methods that could align better with the lifestyles and preferences of PBW [20]. Understanding PBW’s potential acceptability and use of these new options is crucial for designing effective HIV prevention strategies that address their unique needs and challenges.

This study focuses on PBW from South Africa and Botswana, two countries in southern Africa with high HIV prevalence, and multiple public health initiatives aimed at reducing transmission [21–23]. The Health Belief Model (HBM) is a foundational model in health behaviour research, commonly used to understand preventive behaviours across various contexts. We applied the HBM as a framework in this qualitative analysis to explore the attitudes of PBW toward new long-acting PrEP modalities [24, 25]. The HBM theorises that individual health behaviours are shaped by six key constructs: perceived susceptibility to a health threat (in this case, HIV), perceived severity of that threat, perceived benefits of the health action (e.g., taking PrEP), perceived barriers to taking action, cues to action (external or internal triggers to prompt behaviour), and self-efficacy, defined as the confidence in one’s ability to take the desired action. We used the HBM because of its suitability to explore how women assess the risks and benefits of PrEP use during sensitive life stages such as pregnancy and postpartum. The model’s emphasis on individual perception makes it appropriate for examining how women’s own assessments of HIV vulnerability during pregnancy and breastfeeding, coupled with their confidence in managing daily pill use or other PrEP modalities, influence decision-making in the absence of consistent partner or provider support. In addition, the HBM has been applied in previous studies on PrEP acceptability and adherence among women, providing a framework to build on while also identifying areas where women’s lived experiences may extend or challenge model assumptions [26]. We aimed to explore preferences, and potential barriers and facilitators to uptake of each PrEP modality. We emphasise differences between PrEP-experienced/exposed vs. PrEP-naïve PBW, as well as AGYW (ages 18–24) and women over the age of 24. Through these comparisons, we aim to highlight the distinct needs, preferences, and barriers faced by PrEP-naïve PBW versus those with prior experience or knowledge, as well as the unique challenges faced by younger versus older PBW [27].

## Methods

### Research settings and study participants

PBW were recruited from routine antenatal and postnatal services in Cape Town, South Africa, East London, South Africa, and Gaborone, Botswana. In East London and Gaborone, recruitment occurred during routine maternal care services at primary healthcare facilities, and PrEP had not been made available at the time of data collection. Therefore, participants from East London and Gaborone were *PrEP naïve* PBW with limited or no prior knowledge of, or experience with PrEP. In Cape Town, participants were identified through ongoing PrEP delivery programs, where pregnant and breastfeeding women routinely received PrEP counselling and education as part of antenatal and postnatal care [28, 29]. As such, even participants who had not used PrEP were considered PrEP-exposed due to their access to and engagement with PrEP services, and their familiarity with PrEP through these programmes. We recruited both PrEP-experienced participants (who had previously used oral PrEP) and PrEP-exposed participants (who had not used PrEP but were familiar with it through programme exposure). A set script was used for screening potential participants, followed by a brief description of the study. Eligibility criteria included being aged 18 years or older, HIV-negative status, current pregnancy (any gestational age) or postpartum status (up to 3 months), and the ability to provide informed consent. The difference in sampling allowed for a variety of daily oral PrEP experiences and knowledge. Eligible participants were invited to participate, and written informed consent was obtained.

### Data collection

Semi-structured interviews were conducted with participants at the three sites from March-June 2023. Interviews were conducted in each site’s primary languages (isiXhosa and English in Cape Town and East London, and Setswana and English in Gaborone) and were audio-recorded. Trained research assistants facilitated discussions using semi-structured interview guides. Participants were first shown a diagram that explained each modality and then questions were asked about participants’ attitudes and preferences towards the different modalities (see supplementary appendix 1 for the guide and diagram). Interviews were conducted in the chosen languages of each setting (isiXhosa and English in Cape Town and East London, Setswana and English in Gaborone) and audio recorded.

#### Data Analysis

Transcribed and translated interview data were imported into Dedoose software for categorisation by trained research staff. The initial codebook was developed based on the interview guide. Four members of the qualitative research team (JCC, LDV, PV, and AG) agreed on the initial codebook, and coding consensus was reached by two reviewers. We applied thematic analysis following Braun and Clarke’s six-phase framework, which included: familiarisation with the data through detailed reading of transcripts, generating initial codes, grouping themes across coded data, reviewing and refining themes to ensure coherence and distinction, defining and naming themes, and finally summarising the findings [30]. This approach enabled both deductive and inductive coding – deductively using the HBM to guide broad areas of inquiry, while remaining open to inductive identification of themes emerging directly from participant narratives. The analytical domains informed by the HBM included: (1) perceived severity and susceptibility – participants’ HIV risk perception during the pregnancy and breastfeeding period; (2) perceived facilitators and (3) perceived barriers for each PrEP modality; and (4) ‘cues to action’ - support needed by participants for PrEP uptake and continuation and 5) self-efficacy [24]. We also allowed key themes to emerge from the data. We held live analysis sessions via Microsoft Teams, where each member of the research team generated themes in real-time using a virtual whiteboard (Canva) with sticky notes and memos for emerging themes. Themes were then confirmed and finalised with the principal investigator (DJD). Data is presented by PrEP experience (PrEP-experienced/exposed vs. PrEP-naïve) and age (AGYW vs. older women).

## Results

### Demographics

**Fig. 2:**
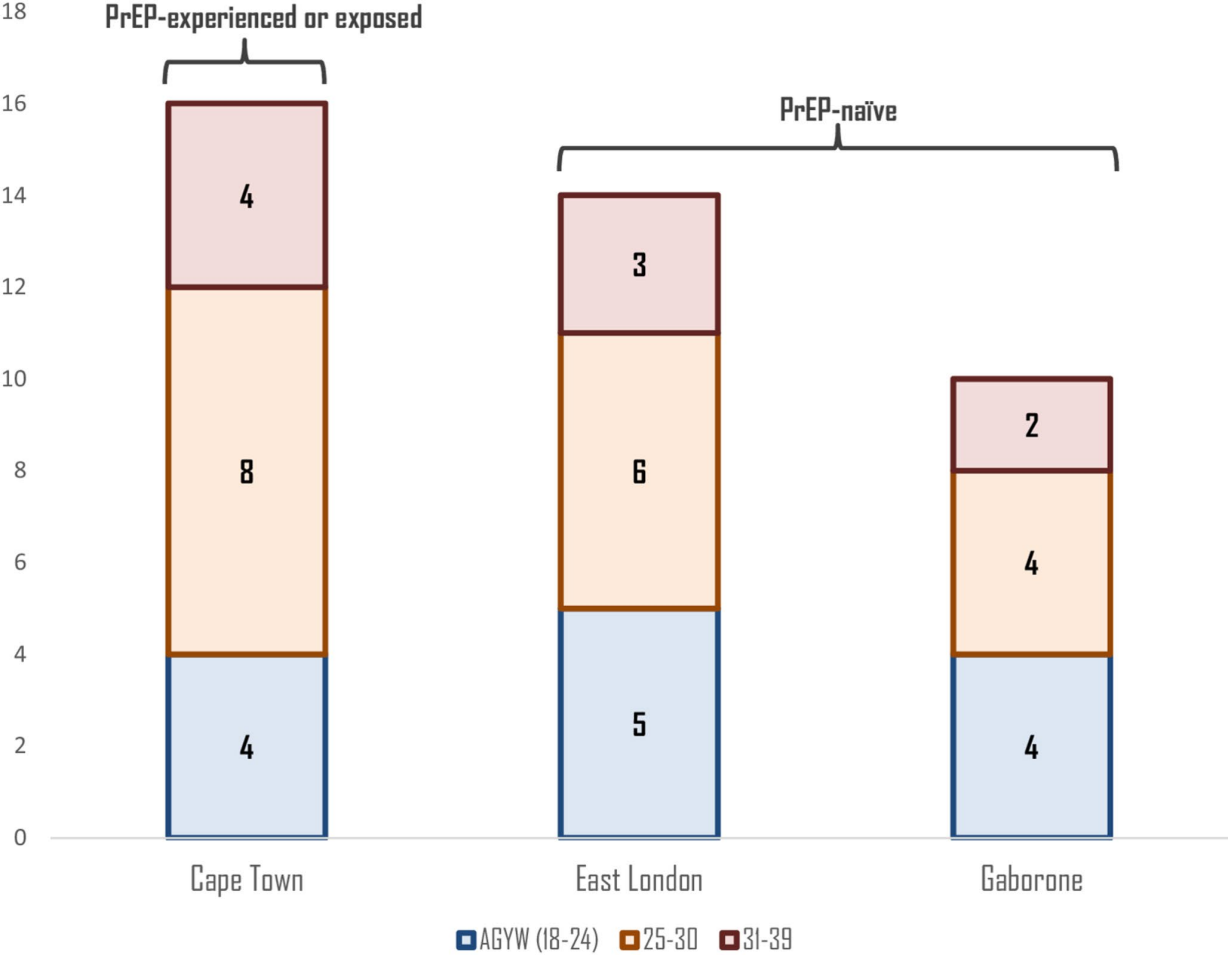
Participants by age group and location

We interviewed a total of 40 participants, aged 18–39 years (mean age: 27 years) (see Fig. 2 for distribution across age and location). Half of the participants were pregnant (*n* = 20), and half were breastfeeding (*n* = 20). The study settings were primarily urban, with all East London participants residing in more peri-urban/rural areas (*n* = 14), while participants those from Cape Town and Gaborone lived urban areas (*n* = 26). All participants in Cape Town were PrEP-experienced (prior use) or PrEP-exposed (no prior use but has good knowledge of PrEP) (*n* = 16), and participants in East London and Gaborone were all PrEP-naïve (no prior use and limited prior knowledge) (*n* = 24). A summary of the perceptions of the different PrEP products and differences by age and PrEP experience can be found in Table 1.

**Table 1:** Summary of perceptions of HIV risk during pregnancy and different PrEP products by age group and PrEP experience

Theme	AGYW (15–24)	Older Women (25–39)	PrEP-Naïve	PrEP-Experienced/Exposed
**Perceived HIV risk during pregnancy**	Pregnancy viewed as a time of vulnerability; motivated to protect baby	Concerns About Partner Infidelity and Non-Disclosure Framed as Broader Health Risks	Limited awareness of PrEP	Recognised HIV vulnerability during pregnancy; motivated by dual protection
**Key Quotes**	*“You know when you are pregnant your body is not as strong to protect itself from other diseases so I thought PrEP might help me to prevent HIV infection”*	*“If you have sex with someone…let’s say your partner is positive*,* didn’t use condom and let’s say he doesn’t take ARVs. That’s where it’s dangerous because you can get infected by HIV*	“*I don’t [know] much about the pill [PrEP]*,* but I have heard people talking about it [but] never seen it or heard what/how it’s used/used for.”*	*“It has affected my decision a lot because I thought to myself that if I get into a risk it will not only be me who is affected so I decided to take it so that I don’t infect an innocent baby so I took a choice to protect it”*
**Monthly Oral PrEP**	Convenience of fewer pills, but concerns raised around access	Convenience appreciated; concerns about forgetfulness	Attracted to potential for fewer side effects; some unfamiliarity with PrEP	Concerns about side effects based on prior use; preferences shaped by experience
**Key Quotes**	*“I would like it to be accessible from the [local] clinic. It must not only be accessible in an area where I have to commute to because I’m unemployed. So*,* I will not have the money to keep on traveling to go get it*,* you see?”*	*“The monthly one you may end up forgetting that you need to go take the pill”*	*“I will take it once*,* for example*,* if I experience any side effects*,* it will be once unlike the daily pill because I will take it every day and keep on reacting.”*	*“If it is making me sick*,* I think it would be difficult to take and continue with it.”*
**Injectable PrEP**	Strong preference due to concerns about forgetting pills and convenience	Interest present, but tempered by past contraceptive experiences and fears of pain	Generally enthusiastic about the reliability and availability	Generally expressed preference, with insights informed by previous experience with contraceptive injections
**Key Quotes**	*“It’s good because some of us forget to take pills on time so the injection would be good.”*	*“The injection is painful”*	*“The injection never runs short in the clinic. I have never heard. Tablets are the ones that are… Injection is something that is not troublesome. You just come to the clinic and get it and go back.”*	*“As long as it will prevent me against HIV*,* I don’t mind because there is no medication with no side effects.”*
**Implant**	More resistant due to fears around insertion and side effects	Mixed views; some valued long-acting, discreet nature and the benefit of not needing to remove the implant as it dissolves	Concerned about pain and side effects; influenced by negative community narratives	Shaped by personal and community contraceptive experiences; some fear of implant-related crime (Cape Town specific). Though others also valued the convenience and long-acting property as it would reduce burden of daily pills
**Key Quotes**	*“I feel that if used…It can change everything in my body. I feel that it might make me a bit sick. It is just risks. It might help somehow but… I feel that it might turn against me on its own.”*	*“Because I will insert it and it will dissolve there will be no need for me to go remove it. It lasts longer than all the other products”*	*“Yes*,* I’m not sure if it’s the one for contraception or this one… Some people say you lose weight when you use an implant. Or you bleed continuously.”*	*“There are many stories people tell about the implant. That when you’ve inserted the implant sometimes*,* you are always dizzy. Or sometimes you lose weight. You gain weight. Or…There are just a lot of stories. So*,* I’m scared of the implant.”*
**Vaginal Ring**	Fears around inserting device into their vagina	Fears framed by prior experiences with contraceptives, which led them to prefer more familiar methods (i.e. pills or injections)	Limited understanding of the ring and the insertion procedure (believing they needed to go to the clinic to have it removed and reinserted every month)	Efficacy of the vaginal ring questioned, which further prevented them from choosing this method which they already considered invasive
**Key Quotes**	*“It would irritate me… There is nothing I like… Because you have to insert it in the vagina. Maybe it gets swallowed inside… I would not be comfortable.”*	*“I once inserted a loop after giving birth…I think one of the reasons I’m sceptical about inserting the ring I saw a string coming out of my vagina so it made me uncomfortable so I had to go to clinic for it to be removed”*	*“Coming here every month to insert and remove it? No. My vagina wouldn’t like that”*	*“No*,* I would not change. You see this one has chances less than 35%? …You would not lean too much on it because the chances of getting HIV are high… And there’s also the fact that it comes on the vagina. Anything to do with the vagina is very sensitive.”*

### Perceived HIV Risk During Pregnancy Shaped Motivation to use PrEP

Participants who were PrEP-experienced/exposed recognised the heightened vulnerability to HIV during pregnancy and were motivated to use PrEP to protect both themselves and their babies. Their narratives often linked pregnancy with physical vulnerability, emphasising a perceived reduced immunity during this period, which made them more receptive to the idea of PrEP for protecting both themselves and child.

*“You know when you are pregnant your body is not as strong to protect itself from other diseases so I thought PrEP might help me to prevent HIV infection.”*

(PrEP-experienced, Cape Town, age 23)

*“It has affected my decision a lot because I thought to myself that if I get into a risk it will not only be me who is affected so I decided to take it so that I don’t infect an innocent baby so I took a choice to protect it.”*

(PrEP-experienced, Cape Town, age 25)

Fear of contracting HIV from partners was a common theme, particularly among older participants who were PrEP-experienced/exposed.

*“It affects my concern very much… as I said he is a person who is cheating so there’s a possibility that I could get it [HIV] and my child could get it too.”* (PrEP-exposed, Cape Town, age 30).

*“If you have sex with someone…let’s say your partner is positive*,* didn’t use condom and let’s say he doesn’t take ARVs. That’s where it’s dangerous because you can get infected by HIV. Sometimes our partners don’t disclose their HIV status… It is dangerous but not if someone can be honest to their partner”* (PrEP-experienced, Cape Town, age 31).

On the other hand, PrEP-naïve participants often displayed a limited understanding of PrEP’s benefits and mechanisms.

*“I don’t [know] much about the pill [PrEP]*,* but I have heard people talking about it [but] never seen it or heard what/how it’s used/used for.”* (PrEP-naïve, Gaborone, age 19).

Overall, pregnancy and breastfeeding influenced participants’ perceptions of HIV risk. Among PrEP-experienced participants, pregnancy was described as a period of heightened vulnerability due to perceived reduced immunity, motivating PrEP use to protect both themselves and their babies. Older PrEP-experienced/exposed participants often contextualised PrEP within broader concerns about partner infidelity and maternal responsibility. They also drew on their familiarity with other prevention methods, such as injectable contraceptives, when discussing PrEP preferences—suggesting that they viewed PrEP as part of an integrated approach to managing their health.

### Long-acting PrEP Preferred for its Convenience,** but Concerns About Pain and Side Effects Persist**

**Fig. 3:**
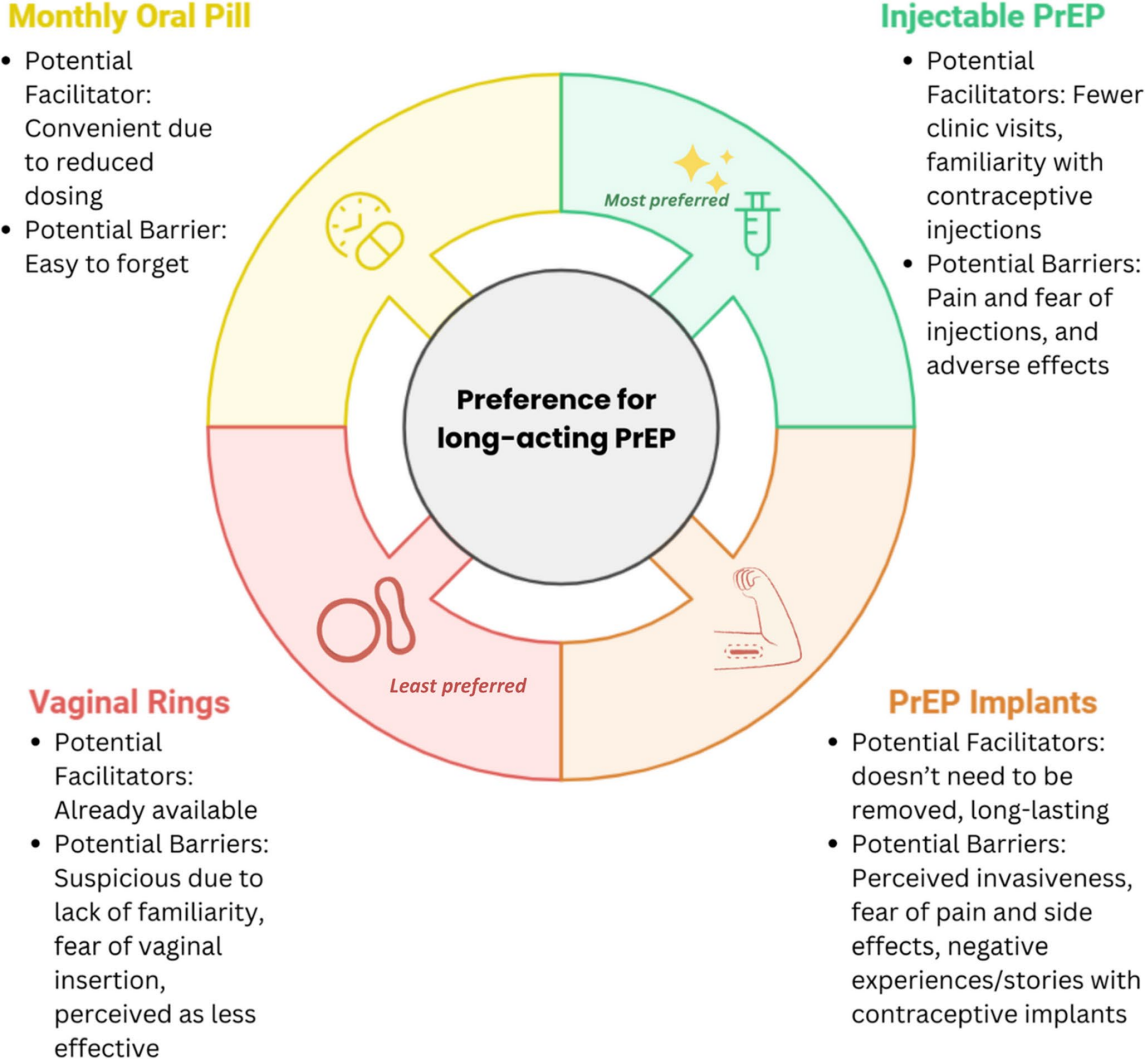
Potential Facilitators and Barriers to each PrEP modality among Pregnant and Breastfeeding Women

There was a clear preference for long-acting PrEP modalities among both younger and older PrEP-experienced/exposed and PrEP-naïve participants. Participants frequently cited perceived convenience due to fewer clinic visits and the daily pill burden as key reasons for favouring these options over daily oral pills (see Table 1; Fig. 3).

### Monthly Oral PrEP Seen as Easier but Concerns Still Raised About Side Effects and Adherence

A predominant facilitator for the acceptance of monthly oral PrEP is the convenience of reduced dosing frequency due to the ease of use, which many participants found appealing.

*“It is the fact that I only take it once. I don’t have to worry about taking a pill every day. I am stress-free for an entire month.”* (PrEP-experienced, Cape Town, age 26).

Both AGYW and older participants also felt that the reduced frequency of administration could lead to fewer side effects.

*“I will take it once*,* for example*,* if I experience any side effects*,* it will be once unlike the daily pill because I will take it every day and keep on reacting.”* (PrEP-naïve, East London, age 23).

However, concerns around barriers to adherence were also significant, with worries around side effects commonly expressed, as well as the dislike of taking pills among a few. Several PrEP-experienced/exposed participants mentioned how adverse reactions, such as nausea and dizziness, would prevent them from continuing taking the pill.

*“If it is making me sick*,* I think it would be difficult to take and continue with it.”* (PrEP-experienced, Cape Town, age 30).

Additionally, the issue of forgetfulness was a common concern across both PrEP-experienced/exposed and PrEP-naïve participants, particularly for older women who were accustomed to daily pill regimens.

*“The monthly one you may end up forgetting that you need to go take the pill”* (PrEP-naïve, Gaborone, age 32).

Barriers surrounding accessibility and socioeconomic factors related to access were also mentioned by AGYW.

*“I would like it to be accessible from the [local] clinic. It must not only be accessible in an area where I have to commute to because I’m unemployed. So*,* I will not have the money to keep on traveling to go get it*,* you see?”* (PrEP-exposed, Cape Town, age 21).

### **Injectable PrEP Seen as Reliable and Convenient, but with Fears Around Pain and Side Effects**

Long-acting injectable PrEP emerged as the most preferred modality among participants, regardless of their PrEP experience and age. AGYW exhibited a strong inclination towards injectable PrEP, primarily due to concerns regarding adherence to daily pill regimens and the convenience of longer intervals between clinic visits. Many expressed the convenience of receiving an injection every two months, which alleviated fears of forgetting to take a daily pill.

*“It’s good because some of us forget to take pills on time so the injection would be good.”* (PrEP-naïve, Gaborone, age 21).

One participant emphasised the reliability of how they find the supply of injections compared to pills, noting the ease of getting an injection at the clinic without concerns about availability.

*“The injection never runs short in the clinic. I have never heard. Tablets are the ones that are… Injection is something that is not troublesome. You just come to the clinic and get it and go back.”* (PrEP-naïve, Gaborone, age 22).

As with younger participants who overwhelmingly preferred injectable PrEP, older PrEP-experienced/exposed participants also generally expressed a preference for injectable PrEP. However, their concerns about side effects were more pronounced. Their insights were often informed by previous experiences with contraceptive methods, revealing a more cautious approach. These made their preferences more complex, as they weighed the benefits of perceived simplicity or convenience alongside potential pain and side effects.

*“As long as it will prevent me against HIV*,* I don’t mind because there is no medication with no side effects.”* (PrEP-experienced, Cape Town, age 29).

PrEP-naïve participants displayed a mix of enthusiasm and apprehension towards injectables. While in support of the modality, they voiced greater concerns about the potential pain involved.

*“The injection is painful”* (PrEP-naïve, East London, age 26).

### Implants Met with Fear and Distrust, but Long-lasting Protection Still Appealed to Some

Participants expressed more resistance than acceptance towards the PrEP implant across both AGYW and older women. Important perceived barriers included fear of pain during insertion, the perceived invasiveness of implant insertion, and distrust in the implant’s ability to dissolve in the body.

*“The implant…Yhoo I am scared of being cut.”* (PrEP-experienced, Cape Town, age 30).

Participants across age groups raised concerns, uncertain about potential side effects, including weight changes, bleeding, and general discomfort or pain.

*“I feel that if used…It can change everything in my body. I feel that it might make me a bit sick. It is just risks. It might help somehow but… I feel that it might turn against me on its own.”* (PrEP-naïve, Gaborone, age 21).

Although some facilitators were mentioned, these were far less frequent, particularly among AGYW. In contrast, older PrEP-experienced/exposed women more commonly appreciated the implant’s long-lasting nature and potential for discreet, low-maintenance use as it dissolves.

*“Because I will insert it and it will dissolve there will be no need for me to go remove it. It lasts longer than all the other products”* (PrEP-experienced, Cape Town, age 29).

Convenience for those who may struggle with adherence to daily PrEP was also mentioned as a potential facilitator. Several participants, despite initial resistance, sometimes changed their preference to implants over daily oral pills despite initial resistance once they understood the long-lasting duration of implants as a preferred benefit of the implants.

*“There’s no need for me to take a pill every day… on that date… at a certain time… I am stress free if I have inserted it. I just need to remember my date.”* (PrEP-exposed, Cape Town, age 26).

### Mistrust of PrEP Implants Rooted in Past Contraceptive Experiences and Community Fears

Personal experiences and community narratives significantly influenced women’s perceptions of PrEP modalities, largely shaped by their prior encounters with contraceptives. Participants were often influenced by stories and external opinions. PrEP-experienced/exposed participants also expressed scepticism towards implants, frequently citing personal experiences of adverse side effects with contraceptive implants, such as weight gain and hormonal imbalances from contraceptive implants.

*“There are many stories people tell about the implant. That when you’ve inserted the implant sometimes*,* you are always dizzy. Or sometimes you lose weight. You gain weight. Or…There are just a lot of stories. So*,* I’m scared of the implant.”* (PrEP-exposed, Cape Town, age 34).

Additionally, some participants voiced concerns about the potential reliability for PrEP implants, recalling anecdotal instances where contraceptive implants were not always effective at preventing pregnancy.

*“I recently had a conversation with my sister and she said it’s not reliable*,* I can still fall pregnant while I have it… and she was not the first person to say that.”* (PrEP-experienced, Cape Town, age 39).

PrEP-naïve participants echoed these sentiments, with many expressing general fears surrounding potential side effects of contraceptive methods, which influenced their views on PrEP. AGYW particularly articulated apprehensions about the impact of PrEP, often shaped by communal narratives of negative contraceptive experiences. This shared concern cultivated a culture of scepticism toward long-acting methods across the age groups.

*“Yes*,* I’m not sure if it’s the one for contraception or this one… Some people say you lose weight when you use an implant. Or you bleed continuously.”* (PrEP-naïve, East London, age 23).

One participant in Gaborone also recounted being injected during a health check-up without fully understanding its purpose, indicating communication gaps from healthcare providers in certain settings around contraception provision that may contribute to scepticism about PrEP implants:

*“[You were injected for?] I do not know. That was the time I was checked. First my womb was checked and was injected saying that they are protecting the baby.”* (PrEP-naïve, Gaborone, age 21).

Participants highlighted the importance of clear understanding of the side effects and uncertainties associated with PrEP, often stemming from lingering concerns from their experiences with contraceptive implants.

*“They can cause pro-longed menses*,* a person might expand (gain weight). Yes. So*,* I would like to know if they would also come and do this to me.”* (PrEP-naïve, Gaborone, age 36).

Uniquely, some PrEP-experienced/exposed participants mentioned fears about criminal activity linked to the contraceptive implant, with concerns that criminals might try to remove the implant for illicit reasons. These worries were especially prevalent among younger women in Cape Town.

*“I don’t like it. Remember criminals will feel that there is something on you and want to fiddle with it and remove it. You see? They remove it! They even remove the current implant [for contraceptives]*,* not the one for PrEP… Apparently*,* it gets smoked. So*,* no.”* (PrEP-exposed, Cape Town, age 21).

### Low Acceptability of PrEP Vaginal Ring due to Unfamiliarity and Discomfort with Insertion

Participants expressed significant concerns regarding the PrEP vaginal ring, primarily centred on the perceived discomfort associated with its insertion and the perceived health risks. AGYW exhibited more pronounced fears surrounding the idea around inserting a device into their vagina, often voicing apprehensions about pain, irritation, or potential complications.

*“It would irritate me… There is nothing I like… Because you have to insert it in the vagina. Maybe it gets swallowed inside… I would not be comfortable.”* (PrEP-exposed, Cape Town, age 18).

Older participants echoed similar concerns, though often framed through prior experiences with contraceptives. Several reported fears that were compounded by negative past experiences with other contraceptives, influenced their preference to opt for more familiar modalities for PrEP options such as oral pills or injections instead.

*“I once inserted a loop after giving birth…I think one of the reasons I’m sceptical about inserting the ring I saw a string coming out of my vagina so it made me uncomfortable so I had to go to clinic for it to be removed”* (PrEP-experienced, Cape Town, age 29).

Participants also questioned the efficacy of the vaginal ring in preventing HIV, which further prevented them from choosing this method which they already considered invasive.

*“No*,* I would not change. You see this one has chances less than 35%? …You would not lean too much on it because the chances of getting HIV are high… And there’s also the fact that it comes on the vagina. Anything to do with the vagina is very sensitive.”* (PrEP-exposed, Cape Town, age 21).

Some participants incorrectly believed that the ring would require insertion and removal by nurses at clinics, which they saw as a significant barrier.

*“Coming here every month to insert and remove it? No. My vagina wouldn’t like that”* (PrEP-naïve, Gaborone, age 30).

Participants’ fears were compounded by their limited understanding of the ring, its usage, and its potential side effects.

*“I do not know the ring*,* I am scared.”* (PrEP-naïve, East London, age 30).

### The Importance of External Support and Self-efficacy for PrEP Adherence

Both PrEP-experienced/exposed and PrEP-naïve participants across both age groups indicated that consistent support would be beneficial for adhering to PrEP methods. Many emphasised the importance of reminders from clinics, family members, or partners, as well as the use of mobile phone alerts.

*“Maybe remind us for example call us and remind us of our next appointment date. I mean they could write a message on the phone to remind you to come on a certain date.”* (PrEP-experienced, Cape Town, age 25).

This support system was seen as essential in helping participants remember dosing schedules, particularly for methods that require regular clinic visits or strict adherence, such as injections or implants.

It is also important to note that although participants acknowledged the valued impact of support from family and partners, both PrEP-experienced/exposed and PrEP-naïve participants expressed a strong sense of agency, emphasising that they would continue taking PrEP regardless of external support, as they prioritised their own health and well-being. This aligns with the concept of self-efficacy in the HBM, as participants displayed confidence in staying motivated to adhere to PrEP on their own.

*“Even if they didn’t support me it wouldn’t have affected my decision because I am doing this for myself*,* this is my health.”* (PrEP-experienced, Cape Town, age 25).

*“[Questions: What will you do if your mother do not support your decision to use PrEP?] I will continue taking it. I want it to protect me.”* (PrEP-naïve, East London, age 23).

### The Importance of Privacy and Discretion,** and Transparency to Break Stigma**

Among older PrEP-experienced participants, PBW reported disclosing their PrEP use with family and partners. This indicated that disclosure fostered a sense of trust and support within their relationships.

*“I am not a secretive kind of a person. I told my family and my partner… The time we first met we did it like that*,* we didn’t just start the relationship and have sex without testing*,* and we were honest from the first start.”* (PrEP-experienced, Cape Town, age 31).

Many PrEP-experienced/exposed participants reported high levels of community awareness and acceptance of PrEP, with older PBW often sharing their experiences more frequently, thereby fostering engagement with healthcare services.

*“People who live in my street know that I am taking PrEP and they ended up coming here at Desmond Tutu [mobile clinic] and joined”* (PrEP-experienced, Cape Town, age 29).

Conversely, younger PrEP-experienced/exposed women exhibited a more cautious approach, often prioritising privacy in their PrEP use, suggesting a desire to keep their PrEP usage private until they felt comfortable sharing.

*“Remember it’s mine*,* not for people*,* right? So*,* it’s fine if only I know about*,* until I feel like disclosing that I have a certain thing that protects me from a certain thing. And then maybe respond when someone needs help to say that there is something like this. [Question: Okay*,* so you would elect for it to be invisible?] Yes”.* (PrEP-exposed, Cape Town, age 26)

This cautiousness was further highlighted by an AGYW who expressed concerns about how her partner would perceive her decision to take PrEP, as well as what reactions there might be from the community. Some AGYW expressed fears of judgment, reflecting the stigma that still exists around PrEP in some communities.

*“Ah I know he will ask questions… ‘why am I taking it*,* am I cheating’ things like that ‘is there something I want to talk about’…as a guy I know he won’t take it as a way I’m taking it. He will think of something else*,* I know for sure… The community that we are living in today it’s like…it’s not a nice place to be.”* (PrEP-exposed, Cape Town, age 24).

However, some PrEP-naïve AGYW also expressed that they would disclose their PrEP use to their partner or family, believing they would receive support.

*“[Questions: Let us say you were taking these pills*,* who do you think you will tell about your decision to use PrEP?] My husband…He will support me”* (PrEP-naïve, East London, age 22).

Additionally, several PrEP-experienced/exposed and PrEP-naïve participants expressed a desire to openly share their use of PrEP to help inform others who might benefit, aiming to break the stigma and promote the protective benefits of PrEP.

*“No I would prefer it to be visible so that if anyone asks me about it I can explain it to them so that they can join if they are interested… So that it can help them as well. What is important is for other people to learn and get help too.”* (PrEP-experienced, Cape Town, age 25).

*“[Question: Do you want it [PrEP] to be something that you can hide?] No*,* other people should know so that they can also get help.”* (PrEP-naïve, East London, age 23).

The findings highlight the nuanced perspectives on privacy, discretion, and disclosure of PrEP use among women of different ages and PrEP experience.

## Discussion

This study explored perceptions of emerging long-acting PrEP modalities among PBW, highlighting how preferences were shaped by prior PrEP experience and age. While oral PrEP was the most familiar option, it was widely described as burdensome due to the requirement of daily adherence, with participants across age groups expressing concerns about remembering to take pills consistently, particularly during pregnancy and postpartum periods when daily routines shift. Injectable PrEP emerged as the most preferred modality overall, valued for its convenience, discretion, and reduced adherence burden. However, concerns about injection pain—especially among younger participants—were notable barriers. Vaginal rings were the least acceptable option across all groups, with women expressing strong aversion due to discomfort about vaginal insertion, fear of the ring moving inside the body, and scepticism about its effectiveness. Implants elicited more mixed responses: younger and PrEP-naïve women voiced fears about side effects and insertion pain, often influenced by negative community narratives, while some older, PrEP-experienced women appreciated the implant’s long-acting, low-maintenance protection. These findings align with those of the subsequent discrete choice experiment, which included *n* = 450 PBW [31]. Findings underscore the importance of modality-specific counselling and tailored education to support informed choice and build confidence in long-acting PrEP options among diverse subgroups of PBW. These subgroup-specific findings informed our subsequent interpretation using the HBM and underpin the tailored recommendations discussed below.

In relation to the HBM construct of perceived severity and susceptibility, our findings suggest that a heightened sense of vulnerability to HIV could motivate PrEP use among PBW, especially among PrEP-experienced/exposed women. Pregnancy was often seen as a period of increased risk, coupled with a strong desire to protect the unborn child. This presents an important opportunity to improve PrEP uptake by aligning messaging with PBW’s existing motivations [32, 33]. PrEP-naïve PBW could benefit from more tailored awareness campaigns on HIV risks, PrEP awareness, and reducing stigma during pregnancy and breastfeeding [34, 35].

Findings based on HBM’s ‘cues to action’ construct emphasised the importance of consistent external support, such as reminders from clinics, mobile phone alerts, or encouragement from family members and partners, in supporting adherence to PrEP [36–41]. These forms of support were considered especially helpful for methods requiring routine clinic visits or time-sensitive dosing, like injectables or implants. However, this external support did not diminish participants’ internal motivation. Both PrEP-experienced/exposed and PrEP-naïve participants articulated a strong sense of personal agency and commitment to prioritising their health, regardless of whether they received support from others. This confidence in their ability to continue using PrEP, even in the absence of external support, reflects the HBM construct of self-efficacy and highlights the dual importance of enabling supportive environments while also recognising the importance of intrinsic motivation among PBW in PrEP use and adherence [42, 43].

At the same time, the nuances seen between AGYW and older women suggest the need for tailored communication strategies to address each group’s specific concerns. AGYW often face barriers to health agency, including limited or negative healthcare experiences, stigma in clinical settings, and peer influences [44–46]. Programmes that promote supportive environments and community-based initiatives could be particularly effective in improving PrEP uptake and continuation among AGYW by offering peer-led support and relatable messaging. Such interventions may also affirm individual agency, thus strengthening both external support systems and internal motivation to sustain PrEP use across age groups [47, 48].

Using HBM’s constructs of perceived facilitators and barriers, this study focused on what influenced PBW’s perceptions and attitudes towards each long-acting PrEP modality. Past contraceptive experiences were found to influence PrEP perceptions, as PBW expressed familiarity and comfort with long-acting PrEP modalities due to previous long-acting contraceptive use, which aligns with other studies [49]. Although many also expressed scepticisms about PrEP implants and injections due to concerns about side effects and effectiveness. Participants often did not make distinctions between PrEP and contraceptive products, and linked side effects with the modality rather than the specific drug. Addressing misconceptions during delivery could help ensure PBW make informed choices based on more comprehensive understanding of each PrEP modality.

Concerns around the invasiveness of options like the vaginal ring and implant highlights the need for a diverse range of PrEP options that accommodate individual preferences. The vaginal ring could benefit from demonstration programmes to increase awareness and comfort with its use. Clarifications also need to be made on the self-insertion of the vaginal ring, as the misconception of having to return to the clinic for removal and reinsertion could prevent uptake.

The demand for discreet, low-maintenance PrEP options demonstrates a global shift toward simplifying HIV prevention interventions. Concerns around privacy and stigma continue to influence decisions regarding PrEP use and disclosure, suggesting that community-based initiatives could provide the social support needed to improve PrEP engagement and reduce stigma [50, 51]. This aligns with cues to action within the HBM, to reinforce sustained PrEP use among PBW [47, 48].

### Implications for Current HIV Prevention Strategies

The preferences and concerns identified in this study provide important guidance for the adaptation of HIV prevention strategies to better meet the needs of PBW, particularly as long-acting PrEP options are rolled out. These findings align with evolving global HIV prevention landscape, particularly as differentiated, person-centred care models gain traction. WHO and UNAIDS have emphasised the importance of expanding PrEP access to underserved populations, including PBW.

This study revealed overall preferences among PBW for long-acting PrEP, which aligns with findings from other research, where similar populations also expressed a strong preference toward long-acting methods due to the reduced pill burden and convenience [49, 52, 53]. There was a particular preference for injectable PrEP, also seen in other studies, which could enhance adherence compared to daily oral regimens [49, 54].

Emphasis will need to be put on the safety and efficacy of long-acting PrEP methods during the pregnancy and postpartum period, especially in healthcare provider training as previous studies have found that healthcare providers have recommended PrEP discontinuation due to fears of the impact on the foetus/child [55]. Misconceptions around the safety and efficacy of long-acting PrEP for PBW could prevent uptake [56]. Furthermore, including PBW in phase 3 studies is crucial to overcoming misconceptions, as it provides robust data on safety and efficacy, thereby fostering informed decision-making and enhancing trust in PrEP interventions.

The individualism observed in this study highlights the importance of offering choices so women in SSA can select the options that best suits their lifestyle best. Providing choices is essential, as life circumstances can change, and women may prefer different options at various times [57]. As new PrEP products, such as the biannual Lenacapavir injectables and three-monthly vaginal ring emerge, as well as the dual prevention pill for HIV and pregnancy, emerge, it will be important to prioritise PBW. Integrating long-acting PrEP into maternal and child health programmes will be vital for addressing the diverse needs of PBW across regions [15, 17, 58]. Ensuring the availability of PrEP options in both urban and rural settings, along with efforts to reduce stigma targeting PBW and AGYW is crucial for fostering better engagement.

### Limitations and Areas for Future Research

While this study provides important insights, its findings are primarily drawn from a sample with a higher proportion of older women and only includes those older than eighteen. Future research should aim for a more balanced representation of younger AGYW to fully capture the spectrum of PrEP perceptions across different age groups. Although different geographical locations have been included, further investigation is needed to explore how regional healthcare infrastructure and cultural factors influence these perceptions, particularly in rural versus urban settings. PrEP-naïve participants lacked familiarity with PrEP, potentially constraining their engagement in detailed discussions about their perceptions and experiences. This contrasts with participants from Cape Town, where participants had more extensive experience with PrEP, enabling richer dialogue.

Finally, longitudinal studies are essential to track how attitudes toward PrEP evolve over time, particularly as new technologies are introduced. Understanding how PBW navigate PrEP choices across different stages of motherhood will be critical for designing timely and relevant interventions.

## Conclusions

In summary, this study underscores the urgent need to understand and address the varied perceptions and attitudes towards PrEP among PBW in SSA. Our findings demonstrate that both age and prior experiences with PrEP significantly influence the acceptance and uptake of various PrEP modalities. Addressing ongoing barriers, especially concerns about the safety and effectiveness of new modalities, could help improve strategies to better assist PBW in utilising long-acting PrEP modalities.

Integrating discussions about PrEP into wider maternal and child health services could also enhance acceptance and uptake. As new PrEP options become available, ongoing research and community engagement will be crucial to ensure that these modalities meet the unique needs of women at different life stages. Ongoing counselling and support will also be crucial for PrEP adherence and continuation as more PrEP options become available. A nuanced understanding of PBW’s experiences and preferences can inform more effective HIV prevention strategies, contributing to global efforts to reduce HIV transmission among the most vulnerable populations.

## Supplementary Information

Below is the link to the electronic supplementary material.

IDI Guide. The in-depth interview guide used for the interviews with participants

## Data Availability

The data that support the findings of this study are available from the corresponding author upon reasonable request. The data are not publicly available due to privacy or ethical restrictions.
