# Correction: Empowering Women’s PrEP Choices: Qualitative Insights into Long-Acting PrEP Preferences and Decision-Making during Pregnancy and Breastfeeding in South Africa and Botswana

**DOI:** 10.1007/s10461-025-04925-2

**Published:** 2025-10-14

**Authors:** Jenny Chen-Charles, Lindsey De Vos, Prisca Vundhla, Avuyonke Gebengu, Elzette Rousseau, Linda-Gail Bekker, Remco Peters, Aamirah Mussa, Chelsea Morroni, Elona Toska, Chibuzor M. Babalola, Jeffrey D. Klausner, Dvora Joseph Davey

**Affiliations:** 1 Desmond Tutu HIV Centre, University of Cape Town, Cape Town, South Africa; 2 Department of Social Policy and Intervention, University of Oxford, Oxford, UK; 3 Desmond Tutu Health Foundation, Cape Town, South Africa; 4 Foundation for Professional Development, East London, South Africa; 5 Department of Medical Microbiology, University of Pretoria, Pretoria, South Africa; 6 Botswana Harvard Health Partnership, Gaborone, Botswana; 7 Usher Institute, The University of Edinburgh, Edinburgh, UK; 8 Centre for Social Science Research, University of Cape Town, Cape Town, South Africa; 9 Department of Population and Public Health Sciences, Keck School of Medicine, University of Southern California, Los Angeles, USA; 10 Department of Epidemiology and Biostatistics, School of Public Health, University of Cape Town, Cape Town, South Africa; 11 Division of Infectious Diseases, Geffen School of Medicine, University of California, Los Angeles, USA

**Correction to: AIDS and Behavior**

10.1007/s10461-025-04856-y

In this article the author’s name Jeffrey D. Klausner was incorrectly written as Jeffrey Klausner.

The original article has been corrected.

