# Supplementary material for: Empowering Women’s PrEP Choices: Qualitative Insights into Long-Acting PrEP Preferences and Decision-Making during Pregnancy and Breastfeeding in South Africa and Botswana
Source: AIDS Behav. 2025 Aug 29;30(1):162–77. doi: 10.1007/s10461-025-04856-y (PMC12816032; doi:10.1007/s10461-025-04856-y)

**Appendix 1: Semi-structured Interview Guide for PrEP-experienced Pregnant and Postpartum Women**

**Introductions:** Hello, my name is ______________________. I am a research assistant and on behalf of Desmond Tutu Health Foundation (or Foundation for Professional Development), I would like to ask you some questions to better understand decision-making about pre-exposure prophylaxis (PrEP) and other methods of HIV prevention among pregnant and postpartum women here in South Africa. In reference to the consent form which you have read/has been read to you, and already signed, do you have any questions for me before we begin?

**Demographics**

- 1. How old are you?
  2. Are you currently pregnant?
     1. If yes, how many weeks?
     2. If no, how long ago did you have your baby?
  3. Was this a planned pregnancy (defined as you were actively trying to get pregnant)?

**Daily oral PrEP use**

- 1. What made you decide to start taking PrEP during pregnancy? *(Probe: did you follow the recommendation of your provider?)*
     1. It can be hard to remember to take a pill every day. In the past three months, how often on average did you take your PrEP? *(Probe: Did you ever stop taking PrEP? If so, for how many days?)*
  2. What factors about your oral PrEP use made it easier for you to continue taking it? (*Probe: what did you like about daily oral PrEP? Did these factors make it easier for you to keep taking PrEP? What have you done to make it easier to take your oral PrEP? Did you use anything to remind yourself to take PrEP?)*
  3. What factors about your oral PrEP use made it harder for you to continue taking it? *(Probe: what did you dislike about daily oral PrEP? Did these factors make it harder to keep taking PrEP? What was your experience getting to the facility for refills?)*
     1. Did you stop taking daily oral PrEP at any point during pregnancy? *(If yes, was it related to these factors mentioned above? When you don’t take it, is it intentional or not? Are there gaps of weeks or months between taking it?)*
        1. *If stopped taking PrEP for any amount of time, is there a specific type of support that might have made it easier for you to continue?* (Probe: peer mentoring, reminder text messages, better education about how to take PrEP)
  4. Did you tell anyone in your life (family, friends, partner, community members) about your PrEP use?
     1. If so, what did they think about your PrEP use? *(Probe: Did they support it? Did they judge you or think lesser of you for using PrEP?)* Did that impact your PrEP use in any way (e.g. how often, where you kept your PrEP, where you took it, etc.)
     2. If not, could you tell me about why you didn’t tell anyone about using PrEP?

**Long-acting PrEP:** There are many types of HIV prevention products being developed, including injections, silicon rings that can be inserted into the vagina, implants that go under the skin and can stay for many months or years and release medication, and pills that can be taken less frequently, such as monthly. Long-acting PrEP means types of PrEP that can be taken less frequently, such as once a month, once every two months, or at even longer intervals. Here are some examples of HIV prevention products and PrEP that are being developed [show participant Table 1 on long-acting PrEP methods in development]. We would like to learn about your preferences when potentially selecting HIV prevention products in the future. For these questions, please assume these long-acting PrEP options would be available to you.

- 1. When thinking about a potential PrEP product, what are the characteristics about it that are most important to you? (*Probe: Examples – few side effects, effective at preventing HIV, frequency, discreetness, frequency of needing to go for refills or clinical appointments, but emphasize that it can be anything)*
     1. Why are those characteristics important to you?
     2. What would your most ideal PrEP product be and why? *(Probes: How would you use it (pill, implant, insert into the vagina)? How often? Would it be visible or invisible?)*

Research is ongoing to develop an injectable version of PrEP that can protect against HIV. This is an injection that is administered in the buttock once **every 4 or every 8 weeks.** Studies show that the injection reduces HIV risk by **almost 90% compared to oral PrEP.** What this means is that women taking injectable PrEP were 9 times less likely to acquire HIV than women taking oral PrEP, which is already very effective in protecting against HIV. In the study, one in every 67 women taking oral PrEP acquired HIV, whereas only one in every 454 women taking the injectable PrEP acquired HIV. The most common side effects were **pain, redness and swelling** at the site of the injection, but very few participants dropped out of the studies due to these reactions. Like oral PrEP, the injection does **not** protect against other STIs or unwanted pregnancy. Studies are ongoing currently to understand the safety of providing a PrEP injection to pregnant and postpartum women seeking to prevent HIV.

- 1. If injectable PrEP was approved as safe for pregnant and postpartum women to use, would you prefer to keep using oral PrEP or switch to the injection?
     1. [If willing or would consider injectable PrEP] What factors about injectable PrEP would make it easy for you to take it?
     2. [If willing or would consider injectable PrEP] What factors about injectable PrEP might make it difficult for you to take it? (*Probe: Do you think it would be hard for you to come back for injections every month or every two months? Why or why not?*)
     3. [If prefers oral PrEP] Why do you prefer oral PrEP over injectable PrEP?
     4. Are there things that you think could help you stay on injectable PrEP long-term (meaning at least one year or more)? *(Probe: Peer mentor or buddy, reminders to go to appointments, being able to get injections close to home or from a local pharmacy)*
  2. Would you tell anyone in your life (family, friends, partner, community members) about your injectable PrEP use?
     1. If so, what do you think they would say about injectable PrEP? *(Probe: Do you think they would support it? Do you think they would judge you or think lesser of you for using injectable PrEP?)*
     2. If not, could you tell me about why not?

Research is ongoing to develop a vaginal ring that can protect against HIV. This is a flexible, silicone ring that is inserted into the vagina and is effective for a full month at a time. Research shows that the ring reduces HIV risk by 35%, meaning that women who used the PrEP vaginal ring were 3 times less likely to acquire HIV than women not taking any PrEP. Because the ring delivers the drug directly to the vagina, side effects may be reduced. Many women in existing studies reported forgetting the ring was in place, and that neither they nor their partner could feel it during sex. Like other forms of PrEP, the ring does not protect against other STIs or unwanted pregnancy. Studies are still ongoing currently to understand the safety of providing the ring to pregnant and postpartum women seeking to prevent HIV.

- 1. If the vaginal ring form of PrEP was approved as safe for pregnant and breastfeeding women to use, would you prefer to keep using oral PrEP or switch to the vaginal ring? Why or why not?
     1. [If willing or would consider the ring] What factors about the vaginal ring would make it easy for you to take it? (*Probes: What about the vaginal ring do you like? Is it more convenient? Easier to insert? Fewer side effects? Other things?)*
     2. [If willing or would consider the ring] What factors about the vaginal ring would make it difficult for you to take it? (*Probe: What about the vaginal ring do you not like? Using it once a month, inserting it in the vagina, no STI/pregnancy protection, other things?)*
  2. Would you tell anyone in your life (family, friends, partner, community members) about using the PrEP vaginal ring?
     1. If so, what do you think they would say about you using the PrEP vaginal ring? *(Probe: Do you think they would support it? Do you think they would judge you or think lesser of you for using the PrEP vaginal ring?)*
     2. If not, could you tell me about why not?

Table 1: Long-acting PrEP methods in development


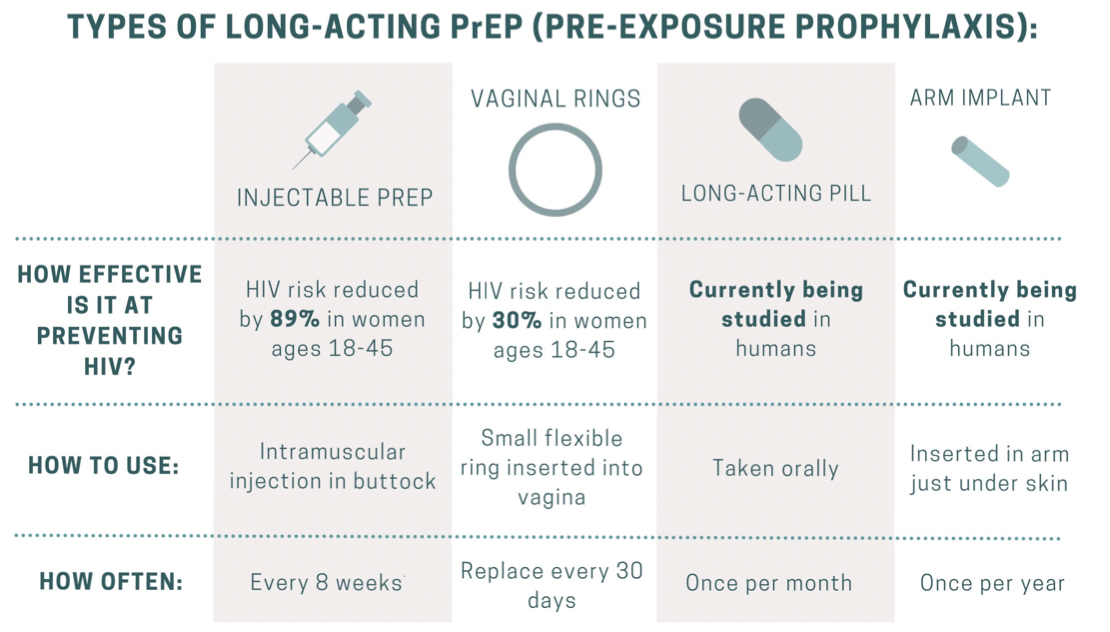

Supplement: Supplementary file 1 — IDI Guide. The in-depth interview guide used for the interviews with participants [file 10461_2025_4856_MOESM1_ESM.docx]
